# A collaborative approach for research paper recommender system

**DOI:** 10.1371/journal.pone.0184516

**Published:** 2017-10-05

**Authors:** Khalid Haruna, Maizatul Akmar Ismail, Damiasih Damiasih, Joko Sutopo, Tutut Herawan

**Affiliations:** 1 Department of Computer Science, Faculty of Computer Science and Information Technology, Bayero University, Kano, Nigeria; 2 Department of Information Systems, Faculty of Computer Science and Information Technology, University of Malaya, Kuala Lumpur, Malaysia; 3 Sekolah Tinggi Pariwisata Ambarrukmo, Yogyakarta, Indonesia; 4 Faculty of Information Technology and Business, Universitas Teknologi Yogyakarta, Yogyakarta, Indonesia; 5 AMCS Research Center, Yogyakarta, Indonesia; Dalian University of Technology, CHINA

## Abstract

Research paper recommenders emerged over the last decade to ease finding publications relating to researchers’ area of interest. The challenge was not just to provide researchers with very rich publications at any time, any place and in any form but to also offer the right publication to the right researcher in the right way. Several approaches exist in handling paper recommender systems. However, these approaches assumed the availability of the whole contents of the recommending papers to be freely accessible, which is not always true due to factors such as copyright restrictions. This paper presents a collaborative approach for research paper recommender system. By leveraging the advantages of collaborative filtering approach, we utilize the publicly available contextual metadata to infer the hidden associations that exist between research papers in order to personalize recommendations. The novelty of our proposed approach is that it provides personalized recommendations regardless of the research field and regardless of the user’s expertise. Using a publicly available dataset, our proposed approach has recorded a significant improvement over other baseline methods in measuring both the overall performance and the ability to return relevant and useful publications at the top of the recommendation list.

## 1. Introduction

The overabundance of information that is available over the internet makes information seeking a difficult task. Researchers find it difficult to access and keep track of the most relevant and promising research papers of their interest [1]. The easiest and the most common approach used in searching for related publications is to send a query message asking the web to provide you with specific information [2]. However, the results from this approach largely depend on how good the user is in fine-tuning the query message beside its inability to personalize the searching results.

Another classical approach used by most researchers is to follow the list of references from the documents they already possessed [3]. Even though this approach might be quite effective in some instances, it does not guarantee full coverage of recommending research papers and cannot trace papers published after the possessed paper. In addition, the list of references may not be publicly available and therefore hard for the researchers to access.

An alternative approach that has been proposed in the literature is the use of research paper recommender systems [4, 5], to automatically suggest relevant papers to the researchers based on some initial information provided by the users that are more elaborate than a few keywords.

To provide more accurate and relevant recommendations, recommender systems incorporate the users’ contexts and the possible contextual information of the consumed contents [6]. Different researchers proposed the use of a different user provided information such as the use of a list of citations [7], the list of papers authored by an author [8], part of paper text [2], a single paper [9], and so on. In these approaches, a user profile is constructed from this initial information to represent the interests of the users and the system search for items or other profiles similar to the one provided to generate recommendations. The challenge was not just to provide a very rich recommendation to researchers at any time, any place and in any form but to also offer the right paper to the right researcher in the right way [10–12].

The major limitation of the existing approaches is their assumption of the availability of the whole content of the recommending papers to be freely accessible, which is not always true due to factors such as copyright restrictions. In an attempt to address this problem, Liu, *et al*. in [3] applied the concept of the collaborative approach to mine the hidden associations that exists between a target paper and its references to provide a unique and useful list of research papers as recommendations.

Motivated from [3], this paper presents a collaborative approach for research paper recommender system. In addition to mining the hidden associations between a target paper and its references, in this paper, we also put into cognizance the hidden associations between the target paper’s citations (see section 3). Similar to [3], our task is not to apply a direct relation between paper-citation relations because, in one way or the other, a researcher who is in possession of a research paper directly or indirectly has access to its limited references and also to its citations. Our aim is to identify the latent associations that exist between research papers based on the perspective of paper-citation relations. A candidate paper is qualified for consideration in [3] if it cited any of the target paper’s references. In our proposed approach, a candidate paper is qualified for consideration if and only if it cited any of the target paper’s references and there exist another paper which cited both the candidate and the target papers simultaneously. We then measure and weigh the extent of similarity between the target paper and the qualified candidate papers and recommend the top-N most similar papers based on the assumption that if there exist significant co-occurrence between the target paper and the qualified candidate papers, then there exist some extent of similarities between them. This strictness in qualifying a candidate paper helps in enhancing the overall performance of the approach and the ability to return relevant and useful recommendations at the top of the recommendation list.

The major contributions of our proposed approach are as follows;

1. We utilized the advantages of publicly available contextual metadata to propose an independent research paper that does not require a priori user profile.
2. Our approach provides personalized recommendations regardless of the research field and regardless of user expertise.

The outline of the rest of the paper is as follows. We first present some related works on recommending research papers. We then detailed our proposed approach. Next, we described our experiments, starting with the dataset and the baseline methods, followed by the evaluation procedures. We then discuss our findings and lastly conclude the paper with a brief concluding remark and future research directions.

## 2. Related work

Research paper recommenders that provide the best suggestions for all alternatives emerged over the last decade to help researchers on seemingly finding works of their interest over the Cyber Ocean of information. Collaborative filtering (CF) is one of the most successful techniques used in recommender systems [13]. It is a method which recommends items to target users based on what other similar users have previously preferred [14–16]. It has been used in various applications such as in recommending movies [17], audio CD [18], e-commerce [19], music [20], Usenet news [16], research papers [7, 21–24] among others (see [25]). Some researchers [13, 21, 26], have criticized the use of this technique to recommend scholarly papers. Precisely the authors in [21, 26], claimed that collaborative filtering is only effective in a domain where the number of users seeking recommendation is higher than the number of items to be recommended, such domains include movies [27], music [28], news [29] etc. While the argument in [13], is that researchers are not willing to spend their valuable time to provide explicit ratings to their consumed research papers, and therefore, leading to insufficient ratings by the researchers to the research papers. Furthermore, for a user to receive useful recommendations, a tangible number of ratings is required.

Nevertheless, despite these aforementioned problems, a significant amount of papers can be traced, which suggest relevant papers to researchers based on collaborative filtering by mining latent associations between scholarly papers. These associations are either directly obtained by taking into consideration paper citations as rating scores [7], or by monitoring the researchers’ actions implicitly [30, 31]. Applying citation analysis such as bibliographical coupling [32] and co-citation analysis [33] has also been used to identify similar papers to a target paper [34].

The relationships among research papers have been categorized into direct and indirect relations in a survey conducted by [35]. In the paper, three approaches were identified for detecting the relationships between papers based on the perspective of paper sources. Namely, citation context, citation analysis, and content-based. The authors claimed that content-based approach becomes less appropriate in detecting relationships across research papers, due to its inability to accommodate some specific characteristics that exist in the research papers like author and citations. Therefore, it becomes suitable only for identifying similarity relations across regular documents. On the other hand, the use of citation analysis can generate more relations between research papers but cannot generate relations from semantic text. This weakness is addressed by using citation context based approach, which depicts more emphasis on determining some important features in the text classification process to increase classification performance.

A context-based collaborative framework (CCF) that uses only easily obtained citations relations as source data was proposed in [3]. The framework employs an association-mining technique to obtain a paper representation of the paper citation context. A pairwise comparison was then performed to compute the extent of similarities between papers. The use of collaborative filtering has also been explored in [7], by using citation-web between scholarly papers to create a rating matrix. The aim was to use the paper-citation relation to recommend some additional references to the input paper. In doing that, the authors investigated the use of six different algorithms for selecting citations. Using offline evaluation, they discovered large disparity in the returned accuracy by each of the six algorithms.

The authors in [6], hypothesized the author’s previous publications to constitute a clear signal of the latent interests of a researcher. The key part of their model was to enhance the user profile with the information coming directly from the references to the researcher’s previous works as well as the papers that cited them. However, the approach increases the well-known sparsity problem. To alleviate this problem, they extend their work in [8], to mine potential citations papers using imputed similarities through the use of collaborative filtering. They also refined the use of citing papers in characterizing a target candidate paper using fragments in the citation and potential citation papers. Whilst the approach works well for researchers with a single discipline, it generates poor results for the multidisciplinary researchers. To overcome this problem, an adaptive neighbor selection approach was proposed in [2], to overcome imputation-based collaborative filtering problem. Whereas authors in [2, 6, 8], recommend papers relevant to the researcher’s interest, they also addressed the serendipitous scholarly paper recommendation in [36].

On another development, the increasing number of research communities and social networking sites such as LiveJournal and MySpace have brought new opportunities for research paper recommendation systems. Researches show that users in online social networks tend to form knit groups [37], with strongly large connected components [38].

Several kinds of research have considered the social group formation and community membership in social networks and their use in recommender systems [39–46]. These researchers utilized the influence of social properties to suggest relevant information to individual or group of users based on social ties, which can either be strong or weak depending on the tie strength that represents the closeness and interaction frequency between the information source and recipient [47, 48].

Recommendations from strong ties are believed to be more persuasive than those from weak ties [49–51]. This is because information transferred by strong ties is likely to be perceived as more relevant and reliable. To be specific, the authors of [45, 52] proposed a novel algorithm called socially aware recommendation of scholarly papers (SARSP) that utilizes the aspect of social learning and networking for conference participants through the construction of relations in folksonomies and social ties. The algorithm recommends research papers issued by an active participant to other conference participants based on the computation of their social ties. This approach has been extended in [53], to include personality behavior in addition to social relations among smart conference attendees. A more detail survey on scholarly data is presented in [54] for more exploration.

The major challenge with the previous researches is that all the contextual information from the recommended, referenced and cited papers must be fully accessible to the recommenders, which are not always freely available due to factors such as copyright restrictions. Another major problem with the existing research paper recommender systems is their dependency on a priori user profile, which makes the system to work well only when it already has a number of registered users, a major hurdle for the construction of new recommender system. Furthermore, the recommendation coverage of most of the current paper recommenders are limited to a certain field of research, this is because recommending papers are stored prior and therefore the system cannot effectively scan the entire databases to find connections between papers. Moreover, most of the existing research paper frameworks are designed to work only on a single discipline, and therefore cannot be used to address the problems of multidisciplinary scholars. While the use of keyword-based query information retrieval technique through search engines is able to scan all document for relevant text, it also provides 100s of irrelevant documents, besides its inability to provide personalize results to the individual researchers.

Different from the existing works, in this paper, we propose a new approach based on collaborative filtering that utilizes only publicly available contextual metadata to personalize recommendations based on the hidden associations that exist between research papers. Our proposed approach does not only provide personalized recommendations regardless of the research field and regardless of user expertise but also handles multi-disciplinary problems.

## 3. Proposed collaborative research paper recommendation approach

Even though some researchers [6, 13, 21, 26], claimed content based to be the most suitable approach when dealing with scholarly domain, other researchers [35] argued on its suitability because only become suitable in identifying similarity relations across regular documents but lacks some important features to effectively detect relationships across research papers.

In this paper, we are motivated to leverage the advantages of collaborative approach as it has proved to be effective in the domains of movies [27], music [28], news [29], e-commerce [19], etc. The unsuitability of the collaborative approach to research paper recommenders was referred to the lack of ratings to research papers by the researchers [13]. In bringing a solution to this problem, we mined rating score between researchers and research papers based on paper-citation relations. We use *C*_*ij*_ to denote citation score between paper *i* and a cited-paper *j* from a paper-citation matrix *C*. If paper *i* cited a paper *j*, *C*_*ij*_ = 1 otherwise *C*_*ij*_ = 0.

We initiate our approach by first transforming all the recommending papers (in our dataset) into a paper-citation relations matrix in which, the rows and the columns respectively represent the recommending papers and their citations. Our approach aimed to deal with scenarios in which: (a) A researcher who finds an interesting paper after some initial searches, wants to get more other related papers similar to it. (b) A student received a paper by his supervisor to start a research in the topic area covered by it. (c) A reviewer wants to explore more based on a received paper that addresses a subject matter which he is not a specialist in. (d) A researcher who wants to explore more from his previous publication(s). In all these cases, we consider a situation where the references and citations of the possessed paper that indicate the user’s preferences are publicly available (which is usually the case in almost all the major academic databases).

**Algorithm 1.** Algorithm representing proposed approach.

Algorithm: Collaborative Research Paper Recommendation

Input: Target Paper

Output: Top-N Recommendation

Given a target paper *p*_*i*_ as a query,

1. Retrieve all the set of references *Rf*_*j*_ of the target paper *p*_*i*_ from the paper-citation relation matrix *C*.
  - For each of the references *Rf*_*j*_, extract all other papers *p*_*ci*_ that also cited *Rf*_*j*_ other than the target paper *p*_*i*_.
2. Retrieve all the set of citations *Cf*_*j*_ of the target paper *p*_*i*_ from the paper-citation relation matrix *C*.
  - For each of the citations *Cf*_*j*_, extract all other papers *p*_*ri*_ that *Cf*_*j*_ referenced other than the target paper *p*_*i*_.
3. Qualify all the candidate papers *p*_*c*_ from *p*_*ci*_ that has been referenced by at least any of the *p*_*ri*_
4. Measure the extent of similarity $W^{p_{i\to }p_{c}}$ between the target paper *p*_*i*_ and the qualified candidate papers *p*_*c*_
5. Recommend the top-N most similar papers to the user.

We accept the user’s query in order to identify the target-paper. Once the target paper is identified, we apply algorithm 1. The algorithm retrieves all the target paper’s references and citations. For each of the references, it extracts all other papers from the web (google scholar to be precise) that also cited any of those target paper’s references. In addition, for each of the target paper’s citations, it extracts all other papers from the web that referenced any of those target paper’s citations (in other words, all the references to the target paper’s citations) and we refer to these extracted papers as the target papers nearest neighbors. For each of the neighboring papers, we qualify candidate papers that are co-cited with the target paper and which has been referenced by at least any of the target papers references. We then measure the degree of similitude between these qualified candidate papers and the target paper by measuring their collaborative similarity using Jaccard similarity measure given by Eq (1). We then recommend the top-N most comparable papers to the researcher.

Jaccard similarity does not only measure the extent of similarity between our target paper and any of the qualified candidate papers but also measures their deviations. Given two papers *X* and *Y*, each with *n* binary attributes, the Jaccard coefficient *J*, is a useful measure of the overlap that *X* and *Y* share with their attributes. Each attributes of *X* and *Y* can be either 0 or 1. The Jaccard similarity coefficient *J*, is given as

$$J=W^{Pi\to Pc}=\frac{Z_{11}}{Z_{01}+Z_{10}+Z_{11}}$$ (1)

where,

*Z*_11_ Represents the total number of attributes where *X* and *Y* both having a value of 1.

*Z*_01_ Represents the total number of attributes where the attribute of *X* is 0 and the attribute of *Y* is 1.

*Z*_10_ Represents the total number of attributes where the attribute of *X* is 1 and the attribute of *Y* is 0.

**Fig 1:**
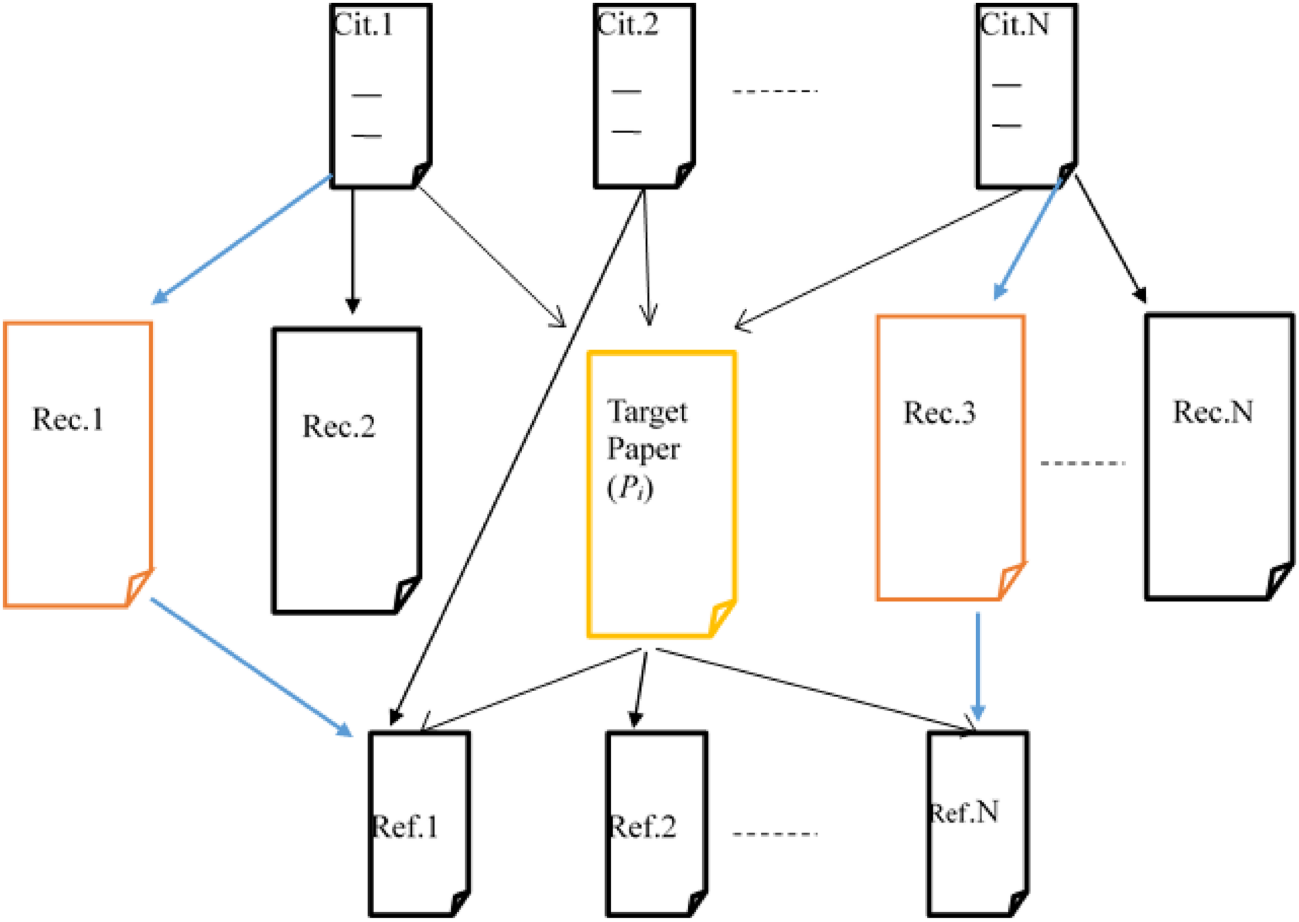
Proposed recommendation scenario.

To illustrate our approach further, Fig 1 represents a target-paper (*p*_*i*_) with references (*Rf*_1_ to *Rf*_*N*_) and citations (*Cit*.*1* to *Cit*.*N*). Each of the references of the target paper has other citations from any of *Rec*.*1* to *Rec*.*N* and/or *Cit*.*1* to *Cit*.*N* other than the target-paper (*p*_*i*_). Also, each of the citations to the target paper has other references from any of *Rec*.*1* to *Rec*.*N* and/or *Rf*_1_ to *Rf*_*N*_ other than the target-paper (*p*_*i*_). Our approach qualifies recommending papers (*Rec*.*1* to *Rec*.*N*) that are co-cited with the target paper and which has been referenced by at least any of the target papers references.

For example, from Fig 1, *Rec*.*1* and *Rec*.*2* are co-cited with the target paper by *Cit*.*1*. However, *Ref*.*2* does not have any connection to any of the target paper’s references and therefore disqualified by step 3 of our proposed algorithm. On the other hand, *Rec*.*1* does not only being co-cited with the target paper by *Cit*.*1* but also referenced one of the target papers references *Ref*.*1*. As can be observed from Fig 1, only *Rec*.*1* and *Rec*.*3* are qualified candidate papers.

In the following section, we present the experiments setup.

## 4. Experiments setup

### 4.1 Dataset

We utilize the publicly available dataset presented in [2]. The dataset contained the publication list of 50 researchers whose research interests are from different fields of computer science that range from information retrieval, software engineering, user interface, security, graphics, databases, operating systems, embedded systems and programming languages. We retrieved every one of their references and citations and extracted from google scholar, every other paper that cited any of the references as well as all the references of each of the target paper’s citations. Some statistics of the utilized dataset is presented in Table 1.

**Table 1:** Statistics of the utilized dataset.

Total number of researchers	50
Average number of researchers’ publications	10
Average number of citations of each researchers’ publications	14.8 (max. 169)
Average number of references to each researchers’ publications	15.0 (max. 58)
Total number of recommending papers	100,351
Average number of citations of the recommending papers	17.9 (max. 175)
Average number of references to the recommending papers	15.5 (max. 53)

### 4.2 Baseline methods

In assessing the effectiveness of our proposed framework, we compare the recommendation results with two baselines presented in [7] and [3]. The pattern introduced in [7] views citation relation matrix as a rating score and generates the recommendation based on common citations between the target paper and its neighboring papers. Given a target paper, the algorithm counts the number of times other citations were co-cited with it. The algorithm then recommends citations with the highest total co-citations summed over all recommending papers. The assumption was that, the more the co-citation in like manner between papers the higher their similarity. While [3], mined the hidden relationship between a target paper and all of its references. The task was to quantify the degree of closeness between the target paper and the other papers that also cited any of the target paper’s references. The rationale behind the approach was that, if two papers are significantly co-occurring with the same citing paper(s), then they should be similar to some extent.

### 4.3 Evaluation metrics

In order to evaluate the quality of our approach, for each of the target papers, we performed 5-fold cross validation to its references and citations by selecting 20% as a test set. We then assess the general performance using the three most commonly used evaluation metrics in retrieval systems: precision, recall and F1 measures. Precision given by Eq (2), measures the capability of the system to reclaim as much relevant research papers as possible in response to the target paper request.

$$precision=\frac{\sum (relevant\_papers)\cap \sum (retrieved\_papers)}{\sum (retrieved\_papers)}$$ (2)

On the other hand, recall given by Eq (3), measures the capability of the system to reclaim as few irrelevant research papers as possible in response to the target paper request.

$$recall=\frac{\sum (relevant\_papers)\cap \sum (retrieved\_papers)}{\sum (relevant\_papers)}$$ (3)

Moreover, F1 measure given by Eq (4) is the harmonic mean between the precision and recall.

$$F1=\frac{2\times precision\;\;\;\times recall}{precision\;\;\;+recall}$$ (4)

As users often scan only documents presented at the top ranked of the recommendation list, we feel imperative to also measure the system’s ability to provide useful recommendations at the top of the recommendation list using the two most widely used ranked information retrieval evaluation measures: Mean Average Precision (MAP) and Mean Reciprocal Rank (MRR).

Average precision (AP) is the average of precision values at all ranks where relevant research papers are found and Mean Average Precision (MAP) given by Eq (5), is the average of all APs.

$$MAP=\frac{1}{I}\underset{i\in I}{\sum }\frac{1}{ni}\sum_{k=1}^{N}P(R_{ik})$$ (5)

Where *P*(*R*_*ik*_) denotes the precision of returned papers from the top until paper k is reached, *N* represents the length of the recommendation list, *ni* is the number of relevant papers in the recommendation list and *I* is the set of papers.

Mean Reciprocal Rank (MRR) given by Eq (6), represents the ranking level at which the system returned the first relevant research paper averaged over all researchers. It measures the extent of the system to return a relevant research paper at the top rank of the recommendation list.

$$MRR=\frac{1}{N_{p}}\underset{i\in I}{\sum }\frac{1}{rank(i)}$$ (6)

Where *rank*(*i*) is the highest ranking where the first relevant research paper *i* appears, and *N*_*p*_ represents the total number of target papers.

## 5. Results and discussions

**Fig 2:**
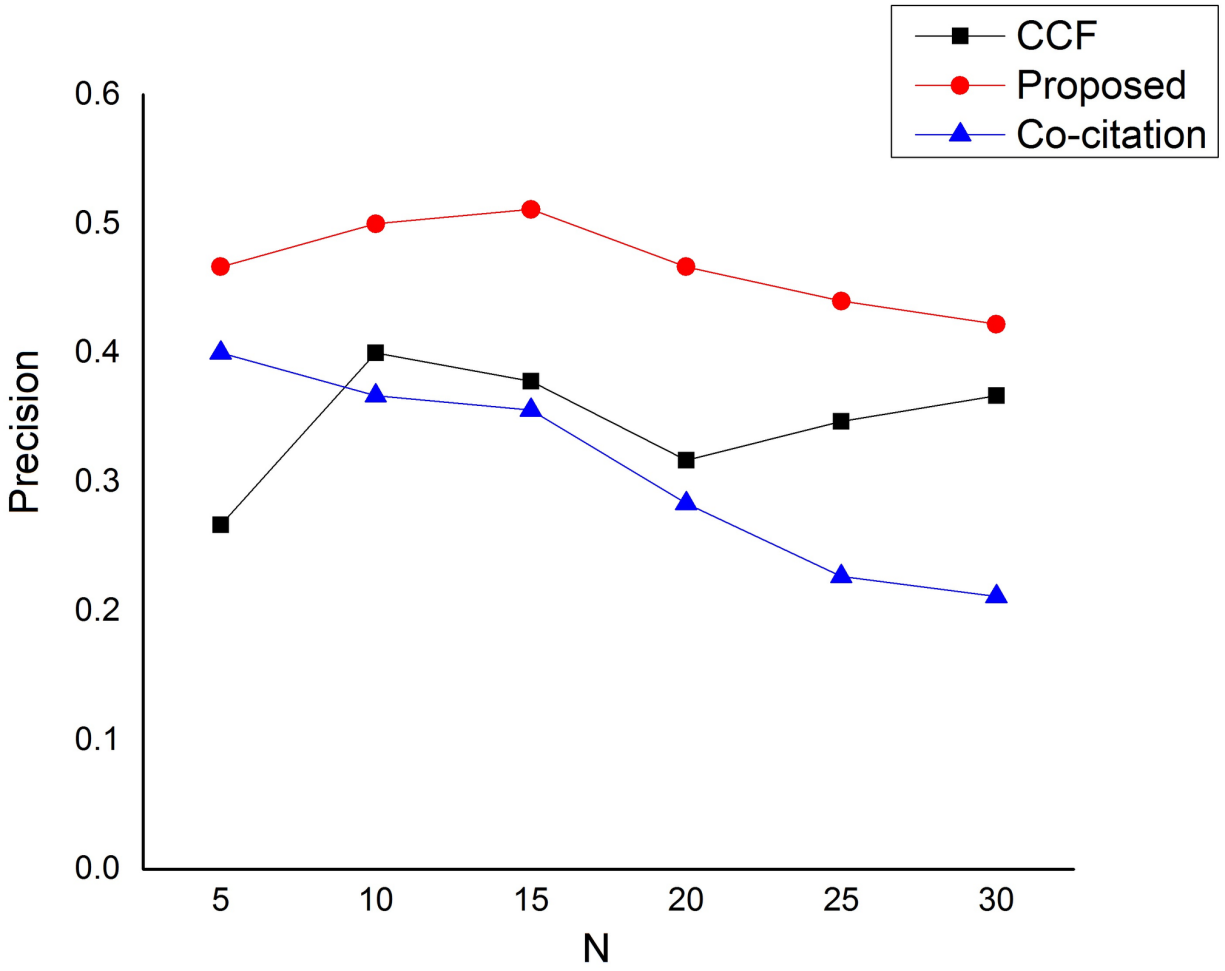
Precision performance on the dataset.

**Fig 3:**
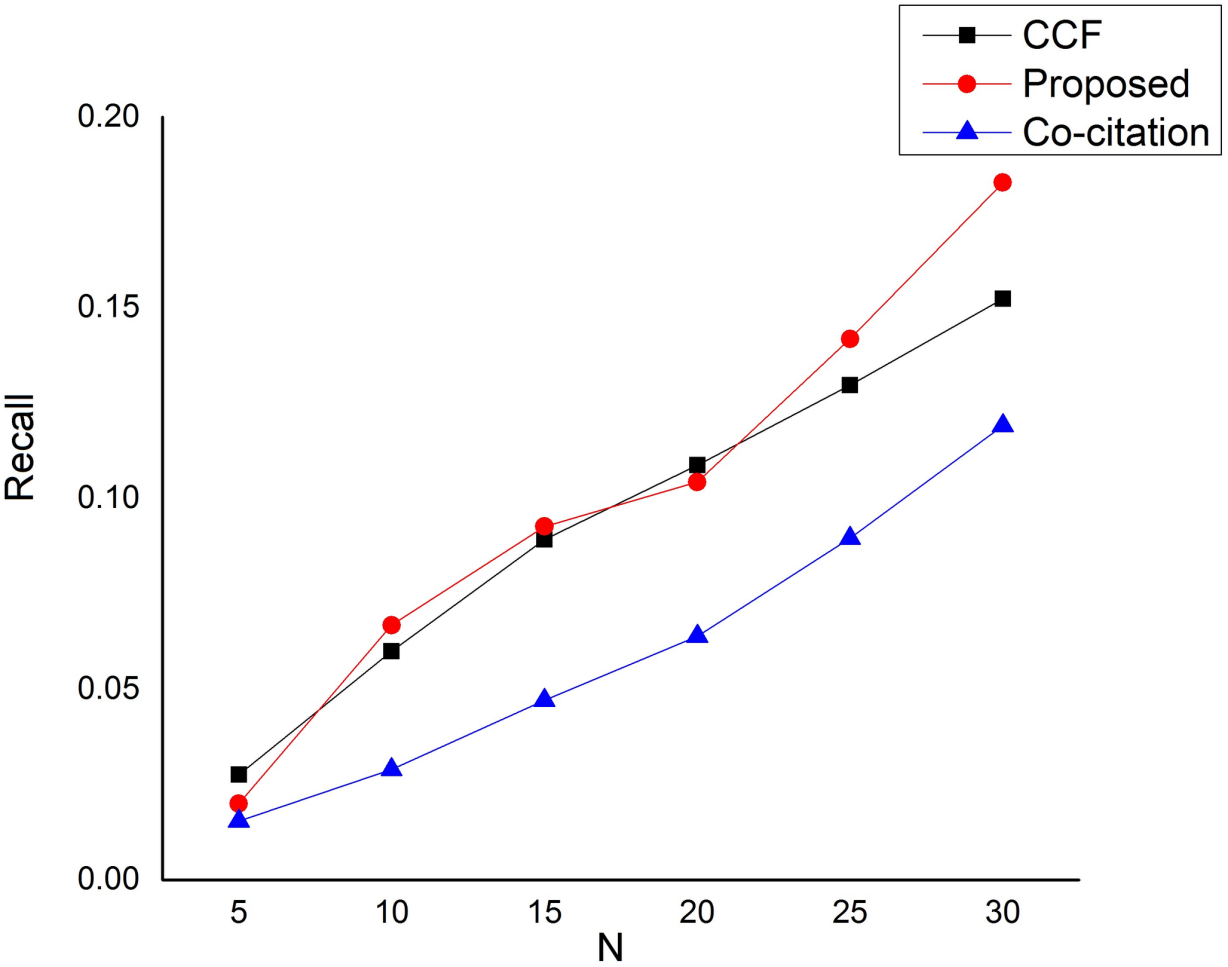
Recall performance on the dataset.

**Fig 4:**
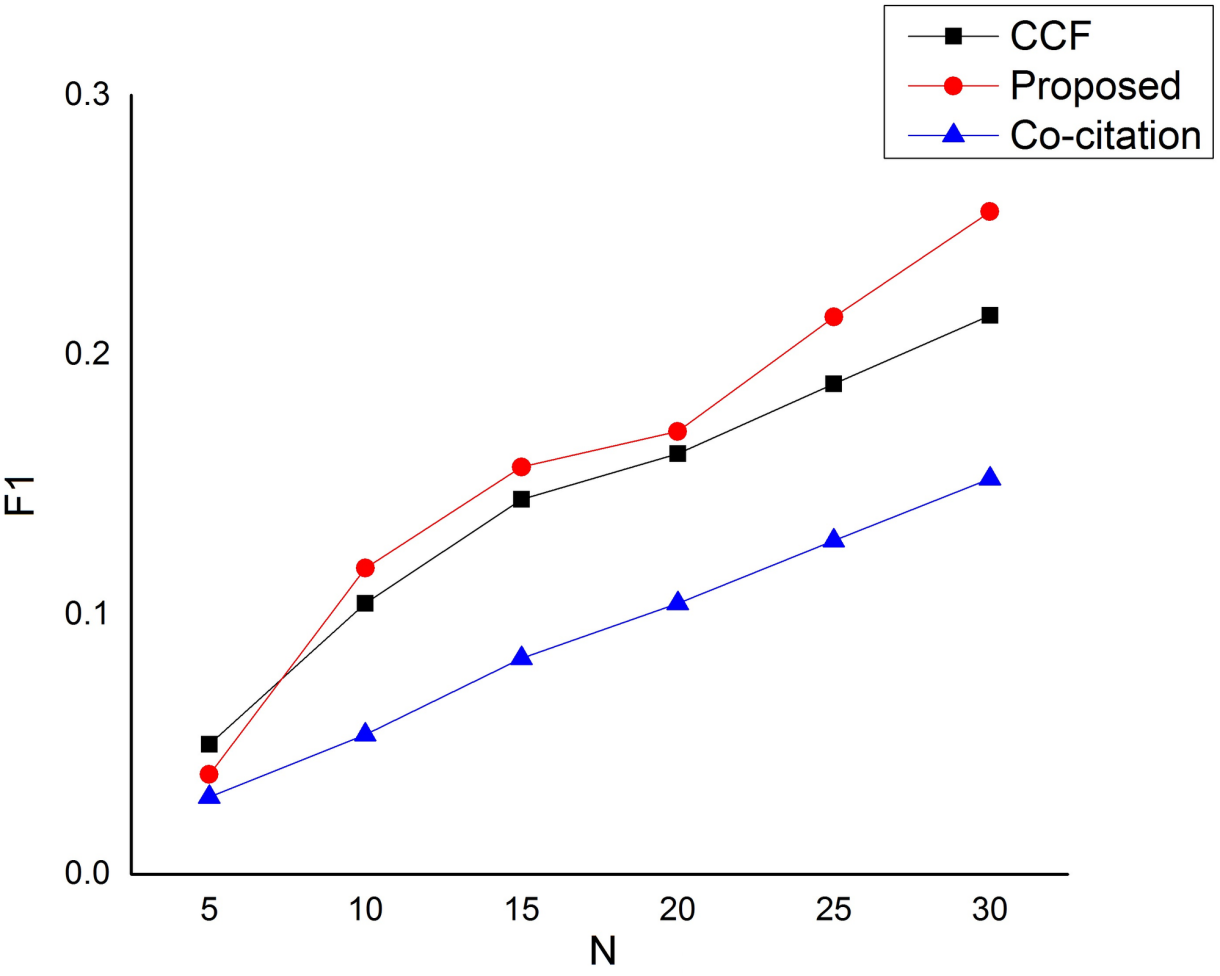
F1 performance on the dataset.

To be specific, the results of each evaluation metric in this section represent the overall averages over all the 50 researchers of our dataset. We start the comparison by assessing the general performance of our proposed approach in returning relevant research papers with the baseline methods based on the three most commonly used information retrieval evaluation metrics. Figs 2–4, demonstrate the comparisons based on precision, recall and F1 evaluation measures respectively. As can be seen from Fig 2, the precision results of our proposed approach has significantly outperformed the baseline methods (Context-Based Collaborative Filtering (CCF) proposed by [3] and Co-citation method proposed by [7]) in returning relevant research papers for all *N* recommendations values. This is because our approach is able to critically remove recommending papers that are less related to the target paper.

Fig 3 depicts the comparison based on recall. As can be seen from the figure, the performance difference between our proposed approach and CCF is very much insignificant. In fact, the CCF method is even slightly better than our proposed approach when *N* = 5 and when *N* = 20. However, our proposed approach began to show the significant difference as the number of *N* increases, specifically when *N* is above 20. The low performance based on recall of our proposed approach is as a result of strict rules in qualifying a candidate paper. Thus, our approach is only after the most significant related recommending papers to the target paper and therefore leaving a lot of other less related papers unrecalled. Furthermore, Fig 4 depicts the harmonic mean between the precision and recall (*F*1 measure), and from the figure, the performance difference between our proposed approach and CCF is also insignificant for values of *N* less than or equals to 20. However, our approach began to show significant improvement over CCF when *N* is greater than 20. In all the three measures, the Co-citation method performs very low compared to our proposed approach. This is because the Co-citation method does not infer the hidden associations between paper-citation relations rather applies direct relations between a target paper and its neighboring papers.

Conclusively, the general performance of our proposed approach has outstandingly outperformed the baseline methods based on precision for all values of *N*. On the other hand, our proposed approach performs worse than CCF in a recommendation list of 5 based on recall and F1 performance measures. However, the major reason behind the low performance of our proposed approach based on recall is the strict rules in qualifying a candidate paper.

Our proposed approach is designed to favor precision which has more influence on user satisfaction than recall. This is because precision is the key element in the process of implementing a search solution [55]. Poor precision damages the reputation of a search system and discourages its use. High precision generally impresses search users [55]. That is why our proposed approach is only after the most significant related recommending papers to the target paper (the result of this can easily be seen from Fig 2), and therefore leaving a lot of other less related papers unrecalled. This is because recall is particularly important in applications where the user cannot afford to miss information such as issues related to security or compliance applications. The recall has less influence on user satisfaction than precision. Many searchers, especially on the Web, are satisfied by precise results, even where recall is low [56]. Notwithstanding, our proposed approach starts to show large disparities with the baseline methods when the number of N is above 5 for both recall and *F*1 measures. Therefore, a very large *N* value is extremely important in order to recall as much qualitative and useful recommendations as possible.

**Fig 5:**
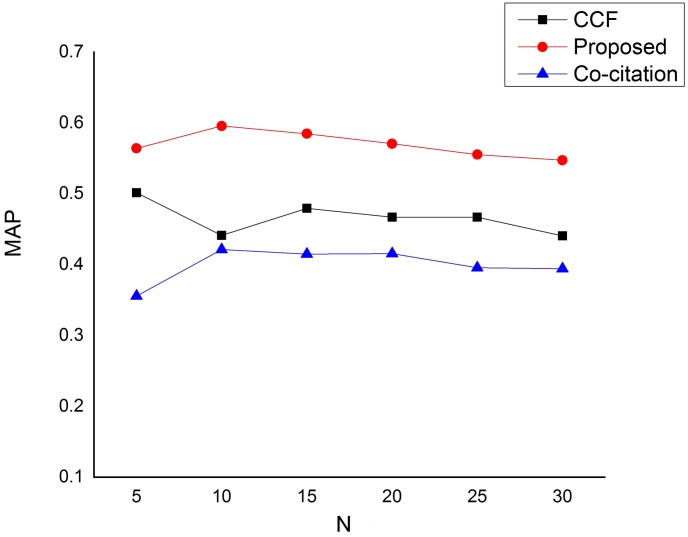
Mean Average Precision (MAP) performance on the dataset.

**Fig 6:**
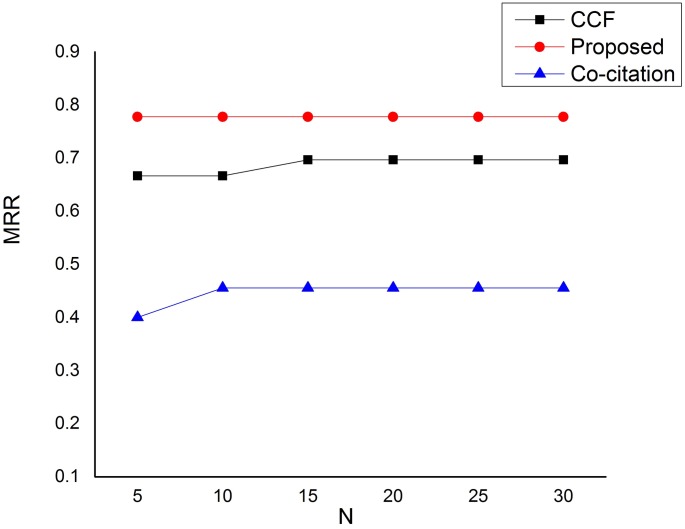
Mean Reciprocal Rank (MRR) performance on the dataset.

Due to the fact that users usually scan only the top of the recommendation list, we also make the comparison based on how our approach is able to return relevant research papers at the top of the recommendation list. Figs 5 and 6 depict our comparisons based on Mean Average Precision (MAP) and Mean Reciprocal Rank (MRR) respectively. As can be seen from Fig 5, that our proposed method has significantly outperformed the baseline methods based on mean average precision (MAP) in all cases in returning the relevant recommendations at the top of the recommendation list. Moreover, the comparison based on mean reciprocal rank (MRR) depicted by Fig 6 has also revealed that our proposed approach has outstandingly outperformed the baseline methods in all scenarios. It can easily be seen from the figure that our approach is able to return a relevant research paper at either rank 1 or rank 2 of the recommendation list for all queries.

As we have pointed out earlier, all these improvements are largely due to the strictness in qualifying a candidate paper which removed less relevant papers to the target paper. This, therefore, increases the system’s ability to return relevant and useful recommendations at the top of the recommendation list.

## 6. Conclusion and future work

In this paper, we utilized the publicly available contextual metadata to leverage the advantages of collaborative filtering approach in recommending a set of related papers to a researcher based on paper-citation relations. The approach mined the hidden associations between a research paper and its references and citations using paper-citation relations. The rationale behind the approach is that, if two papers are significantly co-occurring with the same citing paper(s), then they should be similar to some extent.

As demonstrated using a publicly available dataset, our proposed method outperforms the baseline methods in measuring both the overall performance and the ability to return relevant and useful research papers at the top of the recommendation list. Based on the three most commonly used information retrieval system metrics, our proposed approach have significantly improved the baseline methods based on precision, recall and F1 measures. Our proposed approach has also recorded significant improvements over the baseline methods in providing relevant and useful recommendations at the top of the recommendation list based on mean average precision (MAP) and mean reciprocal rank (MRR).

In addition to considering the collaborative relations among research papers, our next line of research is to also put into cognizance the public contextual contents, such as titles and abstracts of the recommending papers for better performances.

## Supporting information

S1 Dataset The detail of the complete dataset can be accessed via https://figshare.com/articles/Supporting_Information_Dataset_docx/5368408. (DOCX)
