# Supplementary material for: A collaborative approach for research paper recommender system
Source: PLoS One. 2017 Oct 5;12(10):e0184516. doi: 10.1371/journal.pone.0184516 (PMC5628815; doi:10.1371/journal.pone.0184516)
Supplement: S1 Dataset — (DOCX) [file pone.0184516.s001.docx]

# Supporting Information

**S1 Dataset**

The detail of the complete dataset can be accessed via

<https://figshare.com/articles/Supporting_Information_Dataset_docx/5368408>
